# Selective silicate-directed motility in diatoms

**DOI:** 10.1038/ncomms10540

**Published:** 2016-02-04

**Authors:** Karen Grace V. Bondoc, Jan Heuschele, Jeroen Gillard, Wim Vyverman, Georg Pohnert

**Affiliations:** 1 Institute for Inorganic and Analytical Chemistry, Bioorganic Analytics, Department of Chemistry and Earth Sciences, Friedrich-Schiller-Universität Jena, Lessingstrasse 8, D-07743 Jena, Germany; 2 Max Planck Institute for Chemical Ecology, Max Planck Fellow, Hans-Knöll-Str. 8, D-07745 Jena, Germany; 3 Centre for Ocean Life, National Institute of Aquatic Resources, Technical University of Denmark, Charlottenlund Slot, Jægersborg Allé, DK-2920 Charlottenlund, Denmark; 4 Department of Biology, Aquatic Ecology Unit, Lund University, SE-22362 Lund, Sweden; 5 Laboratory of Protistology and Aquatic Ecology, Department of Biology, University Gent, Krijgslaan 281, S8, 9000 Gent, Belgium; 6 California State University, Department of Biology, 9001 Stockdale Hwy, Bakersfield, California 93311, USA

## Abstract

Diatoms are highly abundant unicellular algae that often dominate pelagic as well as benthic primary production in the oceans and inland waters. Being strictly dependent on silica to build their biomineralized cell walls, marine diatoms precipitate 240 × 10^12^ mol Si per year, which makes them the major sink in the global Si cycle. Dissolved silicic acid (dSi) availability frequently limits diatom productivity and influences species composition of communities. We show that benthic diatoms selectively perceive and behaviourally react to gradients of dSi. Cell speed increases under dSi-limited conditions in a chemokinetic response and, if gradients of this resource are present, increased directionality of cell movement promotes chemotaxis. The ability to exploit local and short-lived dSi hotspots using a specific search behaviour likely contributes to micro-scale patch dynamics in biofilm communities. On a global scale this behaviour might affect sediment–water dSi fluxes and biogeochemical cycling.

## Abstract (web summary)

Diatoms often dominate production in aquatic communities, but the amount of available dissolved silicic acid (dSi) limits their growth. Here, Bondoc *et al.*, show that diatoms perceive gradients in dSi, and can increase the encounter with this resource by chemotaxis toward high concentrations under resource-limited conditions.

## Graphical abstract

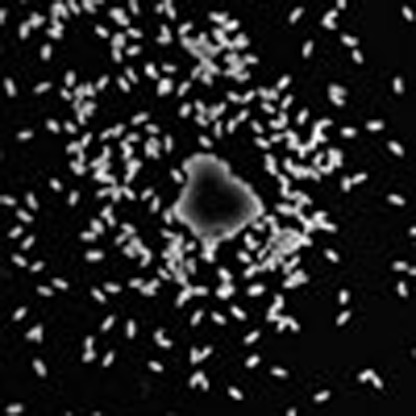

Diatoms contribute about 20% to the global primary production and are key players in marine and freshwater benthic and planktonic communities^1^. A hallmark of diatom physiology is their biomineralized cell wall that is formed by template-catalysed precipitation of silicic acid^2^. Given their vast abundance, diatoms are thereby driving the silicate cycle^3^,^4^. Dissolved silicic acid (dSi) availability is often the limiting factor controlling diatom growth and thus also shaping species composition in marine communities^5^. While the pelagic zone is often dSi-limited below 1 μM, the benthic zone typically shows strong and steep gradients of this resource with higher dSi concentrations (around 150 μM) in the sediment due to the continuous dissolution of deposited minerals^6^. Because of their high productivity and biomineralization activity, benthic diatom biofilms can influence sediment properties^7^ and alter dSi fluxes within the sediment–water interface, thus regulating dSi concentrations in the oceans^8^. Such processes have implications for the transfer of energy to higher trophic levels, bentho-pelagic coupling and hence population and ecosystem productivity^4^. Most benthic diatoms belong to the pennates, comprising the youngest (90 Myr old) yet most species-rich clade within the diatoms^9^,^10^. Many species evolved a strong capacity for vertical migration in sediments under the control of photoperiod and/or tidal cycles^11^. However, these processes alone are not fully explaining observed spatiotemporal dynamics of microbial biofilms, and since many years other factors including the direct and indirect influence of herbivory and microbe–microbe interactions are assumed to guide diatom movement^11^,^12^,^13^. Here we identify an additional guiding factor by showing that diatoms detect and actively move towards dSi sources.

We used the pennate diatom *Seminavis robusta* to explore cell movement and aggregation in response to dSi. Like many other pennate diatoms, this biofilm-forming species moves by gliding through the excretion of extracellular polymeric substances from its raphe, an elongate slit in the cell wall^14^. This allows pennate diatoms to move back and forth. Observed turning movements were suggested to result from the action of extracellular polymeric substance-derived pseudopods or stalks. When a pseudopod or stalk is adhering to the substratum resulting torque supports the whole-cell rotation^15^. In this contribution, we describe three sets of experiments where we first look at the general influence of dSi concentration on diatom motility, then we observe and analyse diatom behaviour in a dSi gradient and last, we test the specificity of the response by comparing the reaction towards dSi and dGe gradients. These experiments clearly demonstrate that diatoms have means to selectively perceive and orient towards the essential resource dSi. A search behaviour in form of increased cell motility and cell speed is observed when the nutrient dSi is depleted. The unicellular algae are also capable of directional movement towards the sources of dSi gradients, a behaviour that supports foraging in the patchy natural environment of benthic diatoms. The fact that structurally closely related dissolved germanium dioxide (Ge(OH)_4_, dGe) sources are not eliciting attraction suggests a specific receptor-mediated response.

## Results

### dSi-dependent diatom motility and speed

**Figure 1:**
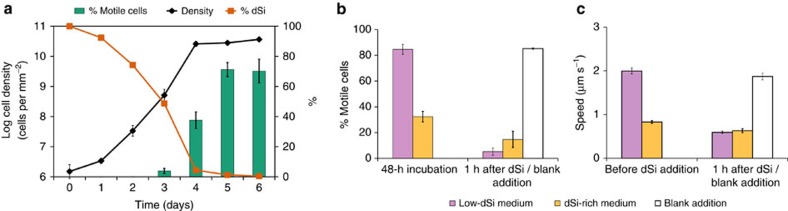
*Seminavis robusta* cell motility is modulated by environmental silicic acid concentrations. (**a**) During silicon (Si)-limited batch culture growth, cell motility increases while dSi (100% dSi=106 μM) is being depleted and the culture enters stationary phase. Error bars show the s.e.m. of five replicates (*n*=300). (**b**) There is a higher percentage of motile cells in low-Si medium than Si-rich medium (one-way analysis of variance (ANOVA), *n*=5, *P*<0.001). After addition of dSi (106 μM) to 48-h silicon-starved cultures, cell motility drops within 1 h (one-way ANOVA, *n*=3, *P*<0.0001). This is not the case after a blank addition of sea water to such silicon-starved cultures (*P*=1.00). Error bars show s.e.m. of three replicates. (**c**) The mean cell speed is significantly higher (LME with Tukey's honest significance difference (HSD) test, *n*=70–200 cells per movie, three movies analysed, *P*<0.0001) in cultures grown in low-dSi medium compared with dSi-rich medium (dSi=246 μM). One hour after the addition of dSi to starved cultures, cell speed significantly dropped (LME with Tukey's HSD, *n*=70–200 cells per movie, three movies analysed, *P*<0.0001) while blank addition did not induce any change on cell behaviour (LME with Tukey's HSD, *n*=70–200 cells per movie, three movies analysed, *P*=0.665,). Error bars show s.e.m. of all tracked cells from three 60-s movies.

To determine if dSi availability affects cell behaviour, we counted motile cells in conjunction with dSi depletion in batch cultures. The proportion of motile cells steeply increased along with decreasing dSi availability as cells entered the stationary growth phase (Fig. 1a). Addition of dSi (106 μM) to stationary-phase cultures elicited within 1 h a marked drop in the proportion of motile cells, indicating a reversible reaction controlled by dSi (Fig. 1b). In addition to motility, cell speed is also dependent on dSi concentration. When stationary-phase *S. robusta* cultures were transferred to artificial sea water without added dSi (low-dSi medium) and were further starved for 3 days, cell movement was more than twice as fast than that of cells transferred to dSi-rich control medium (Fig. 1c). Moreover, cell speed decreased after 1 h of dSi addition while blank addition did not affect the speed. The observed increased proportion of motile cells and higher speed under limiting conditions is a chemokinetic response, that is, a motile response to chemicals. Since dSi is not required for movement, speeding up is an effective mechanism for fast location of this limiting resource during starvation^16^,^17^.

### Directed movement towards dSi sources

**Figure 2:**
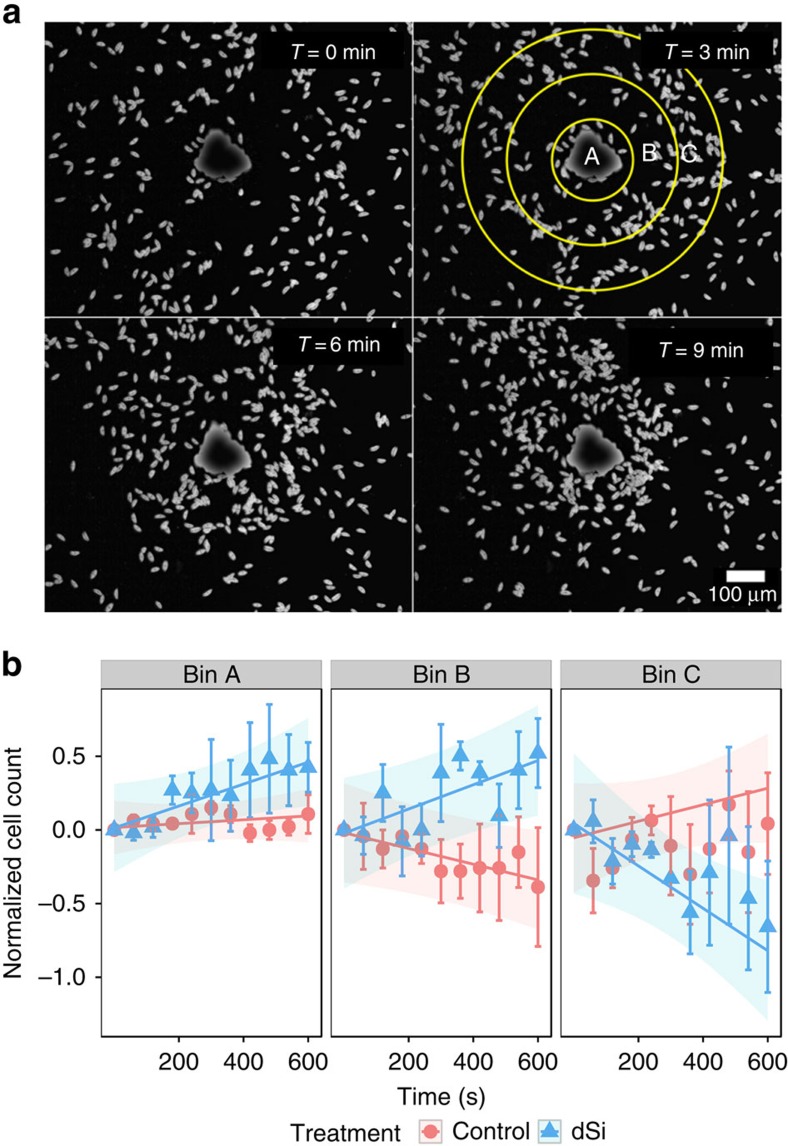
Chemoattraction of dSi-starved cells to dSi-loaded alox beads. (**a**) Light microscopic observation of the cell accumulation (scale bar, 100 μm). The observation area with the alox bead as centre was divided into three concentric rings (Δradius of 112 μm) enclosing bins A–C. (**b**) Plot of the mean normalized cell counts every 60 s, and overlaid shaded area indicating the LME model fit with s.e.m. for each bin (*n*=70–200 cells per movie, three movies analysed). The observed increased cell density in bin B (LME, *P*=0.0034) and decreased cell density in bin C over time (LME, *P*=0.0079) in the dSi treatment reflects the movement of the cells towards the dSi bead.

Since steep vertical and horizontal dSi gradients prevail in benthic environments, an additional strategy to exploit this resource would be a directed movement within dSi gradients. Requirements for such a behaviour are the cells' ability to perceive the resource in a quantitative manner, as described above, and a directed movement towards it. Track analysis revealed that *S. robusta* moves in a back and forth manner that enables cells to reverse direction after each stop (Supplementary Movie 1). This behaviour found in many raphid diatoms and in certain bacteria allows for orientation towards higher concentrations in chemical gradients^18^,^19^. *S. robusta* thus fulfils both above-mentioned requirements for a directed movement. We therefore verified if it indeed has the capability of chemotaxis that would lead to the accumulation of Si-starved cells at local dSi hotspots. Gradients of dSi were generated from micrometer-sized point sources in form of aluminium oxide (alox) beads that were loaded with dSi in different concentrations. To analyse movement along the dSi concentration gradient, the microscopic observation area was divided into three bins (A–C) of equidistant concentric rings around each observed bead covering a radius of 336 μm (Fig. 2a). Since a directed orientation would likely be most relevant under low dSi concentrations found at the water–sediment interface, we aimed to adjust the local concentration gradient in this range. If 1.4 nmol dSi per microscopic bead were applied, ca. 5% diffused out within the 600 s assay period. In a matter of ∼460 s a concentration gradient is established with ∼100 μM dSi at the surface of the bead decreasing to ∼5 μM dSi at the edges of the microscopic observation field (Supplementary Fig. 1). This concentration gradient mimics conditions at the transition of the pelagic and benthic zones^6^. Si-starved *S. robusta* responded to such dSi gradients with an accumulation of cells around the beads while control beads were not attractive (Fig. 2). This behavioural response is observed in different *S. robusta* isolates (Supplementary Movies 2–5). If the observation period is extended to 1 h, continuous movement of cells towards the bead is observed until ∼25 min. After that, chemoattraction becomes less obvious, presumably due to diffusion of dSi. Control beads are not active through the entire assay period (Supplementary Movies 2 and 4). Response to dSi is not limited to *S. robusta*, since *Navicula sp.*, another benthic diatom, also accumulated around dSi sources (Supplementary Movie 6). The administered dSi concentration is in the optimum range to elicit a response. Attraction became less pronounced if lower concentrations of dSi were administered, higher concentrations still resulted in a substantial accumulation around the beads but data became noisier, indicating a more erratic search (Supplementary Fig. 2). Such a concentration-dependent response is typical for receptor-mediated interactions, since higher concentrations of dSi on the beads might cause receptor saturation, whereas lower concentrations fall below the detection limit^20^. The finding capability is thus most efficient in an environmentally relevant concentration range.

**Figure 3:**
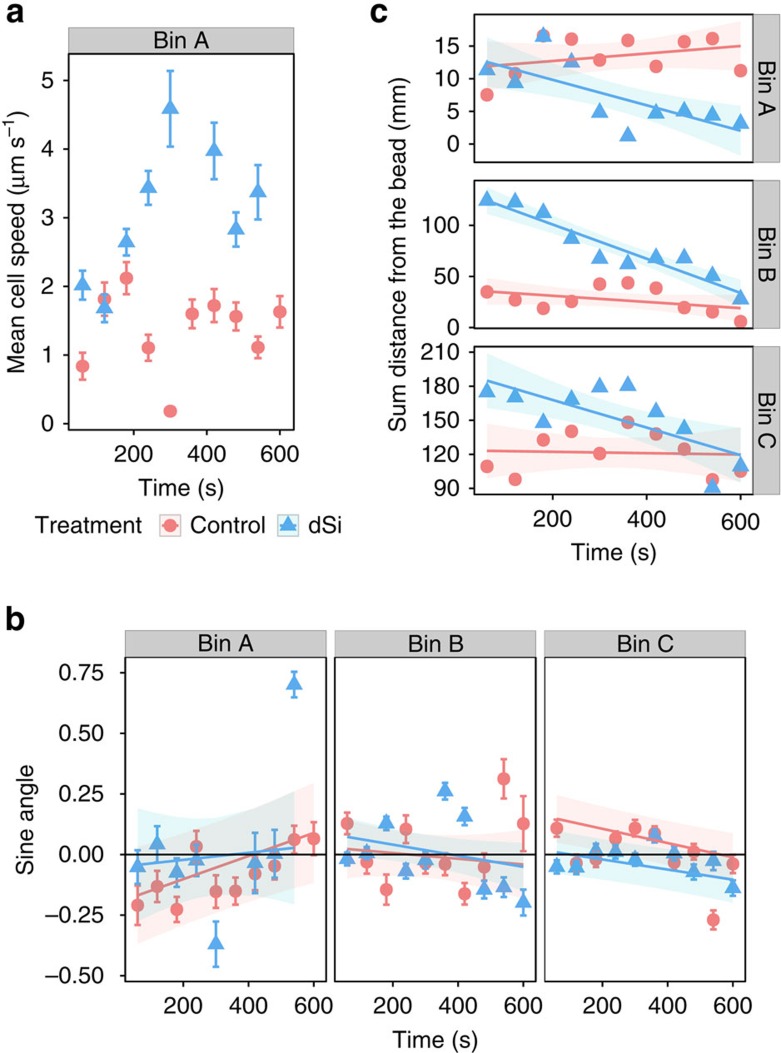
Analysis of track data from dSi and control movies. (**a**) Cell speed increases in bin A (blue triangles) under the influence of the steep dSi gradient. The log+1-transformed mean speed of cells was fitted using generalized additive mixed modelling (GAMM) for each bin. Cells move faster as they approach the bead in bin A (GAMM, *n*_(dSi)_=12, *n*_(Control)_=7, *p*_(Si)_=8.27^−8^, *p*_(control)_=0.176). (**b**) Plot of mean sine angle every 60 s, with overlaid shared area showing LME model fit with s.e.m. Non-directional movement of cells is observed as dSi had no effect on the angular orientation (LME, *n*_(dSi)_=34, *n*_(Control)_=28, *P*>0.05). (**c**) Plot of the sum distance of each cell from the bead every 60 s, with overlaid shaded area showing linear model fit with s.e.m. The decreasing sum distance over time is indicative for the preferential migration of the starved cells towards the dSi-loaded beads in all bins (linear model, *n*_(dSi)_=34, *n*_(Control)_=28, *P*=<0.05).

The fact that starved cells accumulate in the immediate vicinity of the bead suggests that they sense local dSi concentrations and direct their movement up the gradient and ultimately to the source. Surprisingly, the mean swimming speed of cells exposed to a dSi gradient was higher in the close proximity of the beads (Fig. 3a and Supplementary Fig. 3). This only holds true under the influence of the steep dSi gradient in the immediate proximity of the beads and is already not manifested any more in the more distant bin C (Supplementary Fig. 3). While cells generally move faster when starved of dSi (Fig. 1c), it is apparent that an additional response to a steep gradient of dSi is observed. The defined dSi source in an otherwise limited environment causes locally increased speed, which could be the cell's mechanism to avoid diffusion limitation during Si uptake around a dSi hotspot.

### Analysis of movement

The mode of orientation within the dSi gradient was verified in detail by fitting the Taylor's equation^21^ to analyse the swimming characteristics based on tracking individual cell behaviour in response to dSi-loaded and control beads (details in Methods section). *S. robusta* cells have higher directional persistence when perceiving an ascending dSi gradient, as exemplified by longer correlation length and timescales (*λ* and *τ*) (Table 1). Cells within the dSi gradient also had on average a 57% higher diffusivity (*D*) and 81% higher encounter kernel (*β*) for the dSi beads, showing that they maximize their encounter rate to find dSi beads. Analysis of the angular orientation by monitoring the angle of the vectorized tracks relative to the bead centre did not reveal any differences between control and Si-loaded beads, thereby excluding a directed orientation during reversing events (Fig. 3b). However, cells persistently migrate towards the dSi source as indicated by the change in the sum of distances between bead and all cells over time in the treatment compared with the control (Fig. 3c). The observed behaviour can be explained by a preferential forward movement in an ascending gradient of dSi. This directionality during the chemokinetic response thus promotes chemotaxis. The persistent orientation implies a biased random walk, wherein cells adapt their movement patterns to find a dSi source.

### Selectivity of the directed response

**Figure 4:**
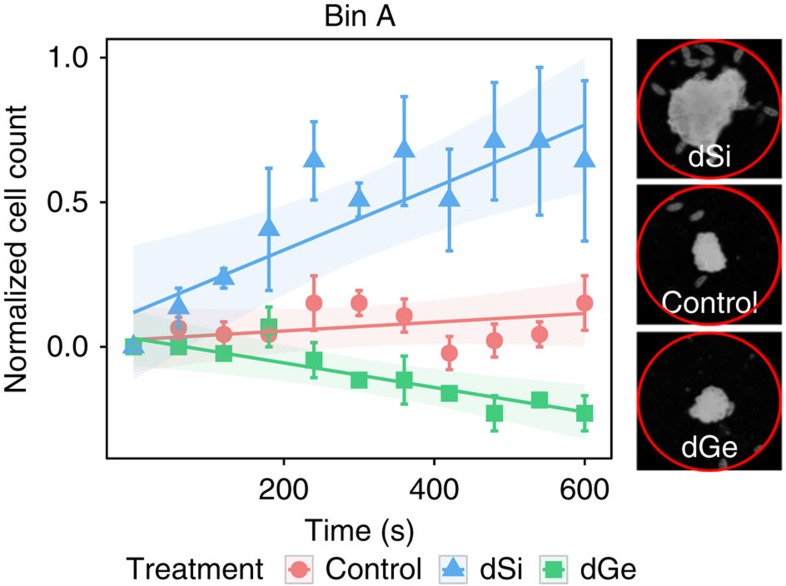
Substrate-specific response of Si-starved cells to dSi- and dGe-loaded (1.4 nmol per bead), and control beads. Plot of mean normalized cell counts with overlaid LME model demonstrates avoidance of starved cells to dGe (LME, *n*=70–200 cells per movie, three movies analysed, *P*=0.0047) and attraction to dSi (LME, *n*=70–200 cells per movie, three movies analysed, *P*=0.0088). Right panels show cells in bin A in selected treatments 600 s after addition of beads (the red circle has a radius of 112 μm).

To learn more about the selectivity of the response to dissolved minerals we determined how Si-starved cells react to dGe sources. dGe and dSi share very similar chemical properties. Ge uptake and incorporation instead of Si in diatom frustules inhibits growth and causes morphological aberrations and toxicity^22^,^23^. When we administered dGe-loaded beads (1.4 nmol per bead) to dSi-starved cultures, a negative response was elicited as cells in average moved away from the dGe sources. In contrast, dSi-loaded beads were attractive, and near constant cell densities were observed around control treatments (Fig. 4 and Supplementary Fig. 4). *S. robusta* thus discriminates between the two very similar inorganic resources. This remarkably specific behavioural response combined with discrimination of the elements during uptake^24^ represents an efficient mechanism to protect the cells against Ge toxicity.

## Discussion

Our results clearly indicate the specific modulation of foraging behaviour of benthic diatoms in response to silicate. A detailed analysis of movement indicates a chemokinetic response since cell speed changes in dependence of dSi concentrations. In addition the observed attraction of cells within a gradient of dSi indicates a chemotactic search capability. Interestingly this search behaviour is not regulated by directed turns of the cells towards the dSi source but rather by a longer directional persistence within an ascending dSi gradient. This finding mechanism thus differs from the chemoattraction mechanism in brown algae where the pheromone-directed movement is mediated by signal molecule-induced turning events of gametes^25^. Such orientation towards dSi or any other dissolved mineral has to our knowledge not been observed before in diatoms. The specificity of the attraction is demonstrated by a selective movement towards dSi while the structurally closely related dGe does not stimulate an attraction response. These observations might be explained by a receptor-mediated process^20^, but until now no candidate receptor mediating a specific recognition of dissolved minerals is known. Since different isolates of *S. robusta* as well as another tested diatom species *Navicula sp.* exhibit this search behaviour, the response might be general for benthic diatoms. The observed dSi-directed movement might thus help to explain the often patchy species composition and structure of marine biofilms.

Tactic behaviour towards nutrient sources such as phosphate and different sources of nitrogen has been demonstrated for other algae and bacteria^26^,^27^. These responses can provide important adaptive advantage for the organisms due to increased acquisition capability for the resources^16^,^17^. The ability of motile diatoms to trace dSi gradients and exploit micro-scale hotspots by changing their foraging behaviour enables them to thrive and dominate phototrophic biofilm communities such as the intertidal microphytobenthos. More general, this capability might be a key factor explaining their explosive radiation in marine and freshwater benthos.

Microbial activity is known to greatly affect global biogeochemical processes involved in the cycling of elements^28^,^29^,^30^. Several mechanisms have been suggested on how microbial behavioural responses to patchy resources can influence ocean biogeochemistry^31^. The ability of diatoms to track dSi availability in the environment has thus implications on a global scale by affecting dSi fluxes and on a micro-scale by shaping biofilm communities.

## Methods

### Cultures

We used the *S. robusta* strains F2-31B and P36 MT^+^ maintained cryopreserved in the BCCM/DCG diatom culture collection at Ghent University (http://bccm.belspo.be/about-us/bccm-dcg)^20^. *Navicula sp.* was isolated from a mudflat at Solana Beach, California 32° 58′ 37.5′′ N 117° 16′ 08.8′′ W. For both species, cells were grown in batch culture^20^ either with natural sea water and F/2 medium^32^ or artificial, buffered sea water (ASW) prepared as described by Maier & Calenberg^33^ to avoid overlaying effects of pH changes due to the treatments. In low-dSi treatments no dSi was added while high-dSi treatments were supplemented with 106 μM dSi for F/2 medium and 246 μM dSi for ASW. Experimental cultures were prepared by 10-fold dilution of aliquot of stock cultures using fresh culture medium and then grown in tissue culture flasks with standard caps, Petri or well plates (Greiner Bio-One, Frickenhausen, Germany). Observations, cell culture photography and video recording were done on an Leica DM IL LED inverted light microscope with a Leica DFC 280 camera system (Heerbrugg, Switzerland).

### Preparation of dSi- or dGe-loaded aluminium oxide particles

Aluminium oxide (100 mg alox, Merck, Darmstadt, Germany; 90 active neutral; 0.063–0.200 mm particle diameter) was used to adsorb silicate by fully evaporating (overnight at 50–90 °C) 800 μl freshly prepared sodium silicate solution (440 mM Na_2_SiO_3_·9H_2_O; Sigma-Aldrich, Deisenhofen, Germany) or germanium dioxide solution (440 mM GeO_2_; Alfa Aesar, Karlsruhe, Germany). To determine the most effective concentration of dSi, 50, 800 and 1,270 μl silicate stock were added to 100 mg alox and evaporated, resulting in ∼0.088, 1.40 and 2.23 nmol dSi per particle, respectively (Supplementary Fig. 2 and Supplementary Table 7). For all succeeding experiments, the concentration 1.40 nmol per particle was used for both dSi and dGe treatments. Blank alox particles for control treatments were identically prepared by evaporation of bi-distilled water. The amount of alox particles per unit weight was determined by counting the number of particles within 10 mg alox beads on tissue culture plates. We counted three randomly chosen microscopic fields (area of each field=5.6 mm^2^) from microscopic photographs. On average 2,640 (±660) particles were present per mg alox.

### Determination of dSi diffusing from the bead

To determine the total flux of dSi diffusing from the bead (*i*), dSi was quantified in water exposed to Si-loaded or control beads after 600 s by standard colorimetric methods^34^. The steady-state concentration of dSi was calculated from the initial concentration on the particle using the formula^18^ *C*_(*r*)_*=i*/4*π√rD*, where *i* is the total diffusive flux of dSi, *r* is the radius where dSi diffused, and *D* is the diffusivity constant for dSi (10^−5^ cm^2^ s^−1^)^35^. Time to steady state was determined as the time ≥*d*^2^/*D* where *d* is the diameter of the whole observation area (672 μm). The *√r* was used to correct the shape of a gradient in a flat chamber^36^. The *i* (1.21^−13^±1.01^−14^ mol s^−1^) was substituted in the equation and the steady-state *C* was calculated based on the distance (radius) from the bead *r*. A plot showing steady-state concentration of dSi against distance can be found in Supplementary Fig. 1.

### Data processing

The open-source software Fiji (http://fiji.sc/Fiji)^37^ with the plug-ins Cell Counter and TrackMate (http://fiji.sc/TrackMate) was used for cell counting and tracking, respectively. All data analyses were done using the open-source statistical and graphic software R version 3.0.3 (http://www.R-project.org/)^38^.

### Motility and speed of *S. robusta* controlled by dSi

For the experiment in Fig. 1a, replicate cultures were grown in Petri dishes (Greiner Bio-One; 60 × 15 mm) in F/2 medium as described above. Five cultures were used for density and proportion of motile cells determinations, performed ∼9 h after the onset of light. In addition, the medium was collected from one more replicate culture each day, by filtration over a 0.2-μm pore size filter and frozen until analysis for silicate content. Dissolved silicate was measured on a spectrophotometer using the molybdosilicate method^39^. For Fig 1b,c, cultures were grown on tissue culture flasks (75 cm^2^ growth surface; filter caps) in 20 ml growth medium with three replicates for each condition. Cultures were grown for 3 days in F/2 medium (106 μM dSi, Fig. 1b) and 7 days in dSi-enriched ASW (246 μM dSi, Fig. 1c). For Fig. 1b, at the beginning of the light period on day 4, the supernatant growth medium was replaced by careful aspiration with a Pasteur pipette attached to a water pump, immediately followed by the addition of ASW with 246 μM dSi or without dSi enrichment. Further culturing was performed for 48 h in continuous light to avoid interference of light–dark alterations on cell motility. Proportion of motile cells was assessed by overlaying two photographs taken at a 15-s interval from the same observation field. Cells (*n*>300) located at the exact same position in both photographs were counted as immotile; others were counted as motile. Cell densities were microscopically estimated by counting cells in at least 35 observation fields with an area of 0.993 mm^2^. The percentage of motile cells was compared before and after addition of dSi (106 μM) or addition of blank artificial sea water without added dSi to cultures grown for 48 h in low-dSi or dSi-rich medium (one-way analysis of variance, Fig. 1b). For Fig. 1c, cell speeds of starved and non-starved cells with addition of bulk dSi (246 μM) or blank addition of artificial sea water without added dSi were assessed by tracking cells from 60-s movies. Differences on treatments were determined by fitting the log+1-transformed speed to a linear mixed effects (LME) model with unique track ID as random factor and a constant variance function structure (varIdent) for treatment. Multiple pairwise comparisons were done through Tukey's honest significance difference (HSD) test (outcome of statistical analysis is given in Supplementary Table 1).

### Movement of Si-starved cells in response to dSi gradients

For all succeeding experiments, *S. robusta* cultures were grown in tissue culture flasks in artificial, buffered sea water with dSi^33^ until they reached stationary phase. On the seventh day, 1 ml of cell suspension was transferred to 12-well tissue culture plates supplemented with 2 ml low-Si medium. Normal light–dark cycle was followed for the 3-day incubation period. Alox particles were carefully administered to each well using a spatula, ensuring that the total number of beads per well does not exceed 30. For obtaining cell count data, photos were taken after exposure to the beads every 60 s for 600 s. Movies for tracking were also recorded for 600 s (1 frame per s). Cell accumulation around the particles was determined from microscopically acquired photographs by counting the number of cells within a circle having an area of 0.300 mm^2^ (for Supplementary Fig. 2) or 0.355 mm^2^ (Figs 2b and 3). For Figs 2b and 4, the observation area was divided into three concentric rings, called bins (bins A–C), having a Δradius of 112 μm with the alox bead as the central point (Fig. 2a).

### Modelling

A representative movie from dSi and control treatments was chosen and cells were randomly selected to be tracked (*n*=29 for control and 34 for dSi) for 600 s. To analyse the track data, cell density (*n*), speed (μm s^−1^), angular orientation (sine angle) and distance (μm) of cells relative to the bead were taken as parameters. Mixed models were used to analyse and fit the data to be able to account for the nested and longitudinal design of the study. Linear modelling of sum distance and LME modelling of cell count and angular orientation were done using the R package *nlme*^40^ while general additive mixed modelling of cell speed was done using the R package *mgcv*^41^. To correct correlated data between independent variables, a correlation structure, autoregressive order 1 (AR-1) was used. A constant variance function structure (varIdent) was also added to the model for correcting residual spreads. Individual models for each bin were chosen based on the Akaike information criterion. For each model, a Wald test was performed to determine the significance of the fitted estimates on each term. All results are shown in Supplementary Tables 2–6.

### Cell counts

Cell counts were standardized according to standard *Z*-score calculation per treatment: standard score *Z*=(*X*−*μ*)/*σ*, where *μ* is mean, *X* is score and *σ* is s.d. For the starting point to be normalized to 0, we subtracted the standardized cell count on each time point to the value at *T*=0 s. A value of 0 indicates that the cell density is equal to the mean. Positive values indicate a cell density higher than the mean and a negative value the opposite. To compare control and dSi treatments (Fig. 2b and Supplementary Table 2), a model for each bin was fitted using the interaction between treatment and time as independent variables and replicate ID as a random factor. An AR-1 correlation structure for successive measurements within the replicates and a varIdent variance structure for treatment on bins A and B and replicates for bin C were added to the model. For the comparison of substrate specificity (Fig. 4, Supplementary Fig. 4 and Supplementary Table 6), the model for each bin was fitted the same way as described above and a varIdent variance structure for treatment was added for all the bins.

### Cell speed

A log+1 transformation was used to normalize cell speed. The mean speed of the cells every 30 s for each bin was fitted using general additive mixed model (Fig. 3a, Supplementary Table 3 and Supplementary Fig. 3). Data for a time point were excluded when only a single-track data contributed to the mean. The independent variable was fitted with a simple factor smooth and penalized with cubic regression splines of time on each treatment, and track ID (that is, unique cell ID) was assigned as a random factor. An AR-1 correlation structure between treatment and track ID was added to the model. In addition, a varIdent structure on treatment was used in the model to decrease the Akaike information criterion significantly and for a better fit.

### Angular orientation

To determine angular orientation, the sine angle of each coordinate position of the cell relative to the coordinate position of the bead was calculated^42^. A cell is moving towards the bead if it has a positive value and away from it in case of a negative value. Average of the sine angles was determined per bin every 60 s (Fig. 3b and Supplementary Table 4). Data for a time point were excluded when only a single-track data contribute to the mean. Each bin data were fitted via LME using the interaction between treatment and time as independent variables and replicate ID as a random factor. An AR-1 correlation structure was used between treatment and replicate ID as well as a varIdent variance structure for treatment.

### Sum distance

The sum of distance from each cell's coordinate position relative to the bead centre was used to determine the migration pattern of cells. Each bin was fitted using a simple linear model (Fig. 3c and Supplementary Table 5) with sum distance as the response variable and the interaction of time and treatment as explanatory variable.

### Motility parameters

To determine whether the motility characteristics between the control and dSi treatment were different, we analysed track data from two representative movies. Motility parameters were computed by fitting the root mean square of the net distance as a function of time to Taylor's equation using nonlinear least-squares estimation^21^: root mean square=[2*v*^2^*τ* (*t*−*τ* (1−*e*^−*t*/*τ*^)]^0.5^ where *v* is effective swimming speed, *τ* is the decorrelation timescale, *t* is the time and *λ* is decorrelation length scale: *λ*=*vτ*.

The decorrelation length (*λ*) and timescale (*τ*) give the distance and time, respectively, wherein there is directional persistence in motility over a period of 600 s.

We also calculated the effective diffusivity of motility (*D*=*v*^2^*τ*/*n*) and encounter kernel (*β*=4*πRD*), where *n* is number of dimensions and *R* is radius of the bead. *D* describes the spread of the cell tracks and *β* determines the water volume screened by cells within the observation time^21^.

## Additional information

**How to cite this article:** Bondoc, K. G. V. *et al.*, Selective silica-directed motility in diatoms. 7:10540 doi: 10.1038/ncomms10540 (2016).

## Supplementary Material

Supplementary Information Supplementary Figures 1-4 and Supplementary Tables 1-7.

Supplementary Movie 1 The movie shows the characteristic back and forth movement of Seminavis robusta (strain F2-31B) synonymous to the run-reverse movement of marine bacteria. Movie was accelerated 10x. Time labels denote min:s.

Supplementary Movie 2 The movie shows the response of S. robusta (strain F2-31B) to a control bead (no dSi). dSi-starved cells move randomly around the area and show no response towards the bead. The video speed was accelerated 50 times and the scale bar indicates 50 μm. Time labels denote min:s.

Supplementary Movie 3 The movie shows the attraction and accumulation of S. robusta (strain F2-31B) to a dSi-loaded bead. dSi-starved cells began to move towards the bead in less than 300s, indicating that they perceived a gradient of dSi diffusing from the bead. The video speed was accelerated 50 times and the scale bar indicates 50 μm. Time labels denote min:s.

Supplementary Movie 4 The movie shows S. robusta (strain P36 MT+) starved and exposed to a control bead for 1h. No response towards the bead was observed for the whole observation area. The video speed was accelerated 50 times and the scale bar indicates 100 μm. Time labels denote min:s.

Supplementary Movie 5 The movie shows S. robusta (strain P36 MT+) starved and exposed to a dSi-loaded bead for 1h. Attraction towards the bead for the whole observation time was evident indicating that the created diffusion gradient was stable for 1h. Furthermore, cells were observed to stop moving around ~25 min which was also observed for dSi-starved cells after bulk addition of dSi. The video speed was accelerated 50 times and the scale bar indicates 100 μm. Time labels denote min:s.

Supplementary Movie 6 The movie shows the attraction and accumulation of Navicula sp. to a dSi-loaded bead for 20 min. Like S. robusta, this pennate diatom also has a back and forth movement. dSi-starved cells also accumulated towards the bead in less than 300s. The video speed was accelerated 50 times and the scale bar indicates 100 μm. Time labels denote min:s

## Figures and Tables

**Table 1:** Motility parameters of cells exposed to control and dSi beads.

**Parameter**	**Control**	**Si**
*N*	28	34
*τ* (s)	4.68	14.85
*λ* (μm)	10.70	23.86
*D* (μm^2^ s^−1^)	12.22	19.17
*β* (μm^3^ s^−1^)	8,200	14,900

Calculated using Taylor's equation, where *N* is number of tracks, *τ* is decorrelation timescale, *λ* is decorrelation length scale, *D* is diffusivity and *β* is encounter kernel (observation area: bins A–C; observation time: 600 s).
