# Supplementary material for: Selective silicate-directed motility in diatoms
Source: Nat Commun. 2016 Feb 4;7:10540. doi: 10.1038/ncomms10540 (PMC4742965; doi:10.1038/ncomms10540)
Supplement: Supplementary Information — Supplementary Figures 1-4 and Supplementary Tables 1-7. [file ncomms10540-s1.pdf]

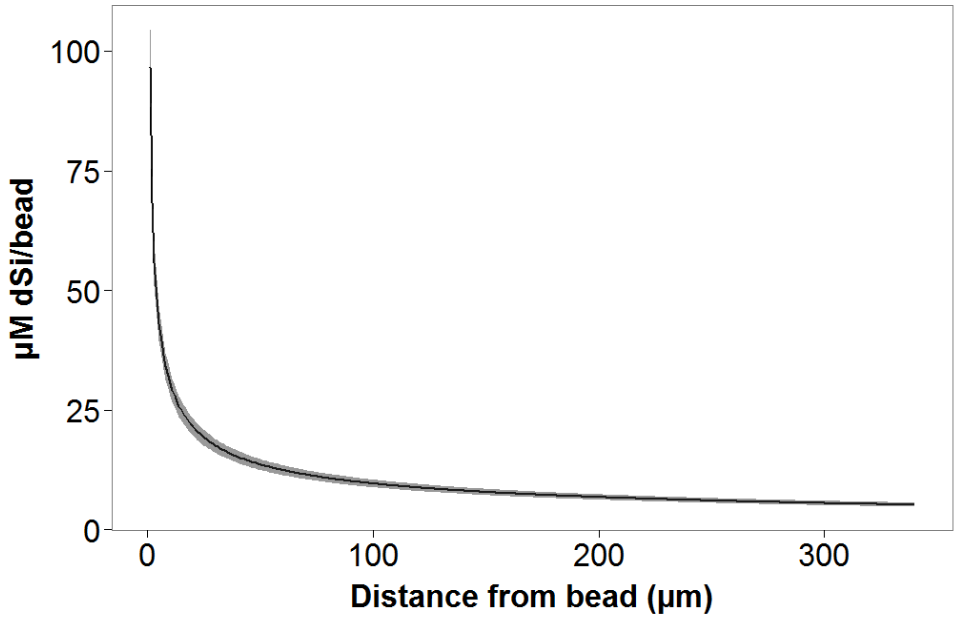

Supplementary Figure 1. Steep dSi concentration gradients that mimic the sediment-water interface are built up around the dSi-alox loaded beads. The gradient depicted here is reached at ~460 s. dSi concentrations in Bin A showed a sudden drop from 97 µM dSi to 9.1 µM while Bins B and C showed a steadier dSi concentration gradient (9-5 µM dSi). dSi was determined in water exposed to Si-loaded or control beads after 600 s on 3 replicates and the concentration depicted here was calculated as described in the methods section. Note: Since beads are not spherical the distance from the bead surface is considered.

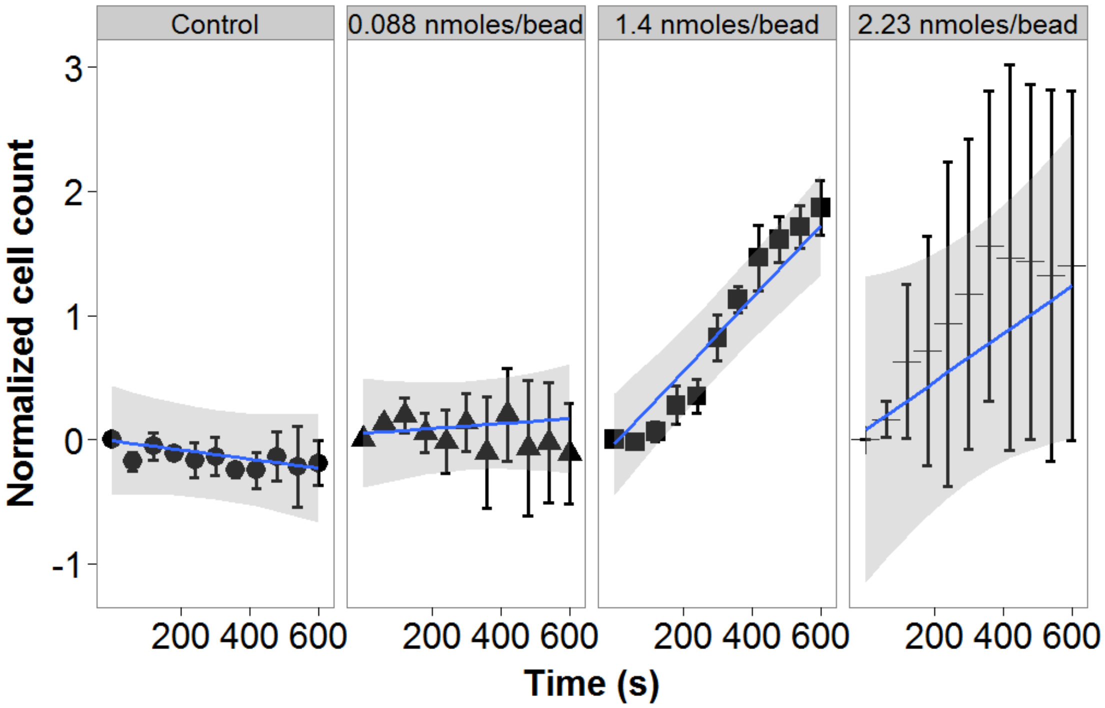

Supplementary Figure 2. dSi-starved cells were exposed to different concentrations of dSi and cell density was determined every 60s in an area of 0.3 mm^2 corresponding roughly to the area of bins A-C (0.35 mm^2). Plotted normalized cell counts from every 60s photos were fitted using a linear mixed effects model and an overlay of the model with SE is shown here. For normalization of the starting point to 0, we subtracted the standardized cell count on each time point to the value at T=0s. A value of 0 indicates that the cell density is equal to the mean. Positive values indicate a cell density higher than the mean. A concentration (1.4 nmoles dSi/bead) that elicited the highest chemotaxis as compared to the control and the other two concentrations ($p=<0.0001$, estimate=0.0033, s.e. =0.00059, DF=116, t=5.62) was used for further experiments.

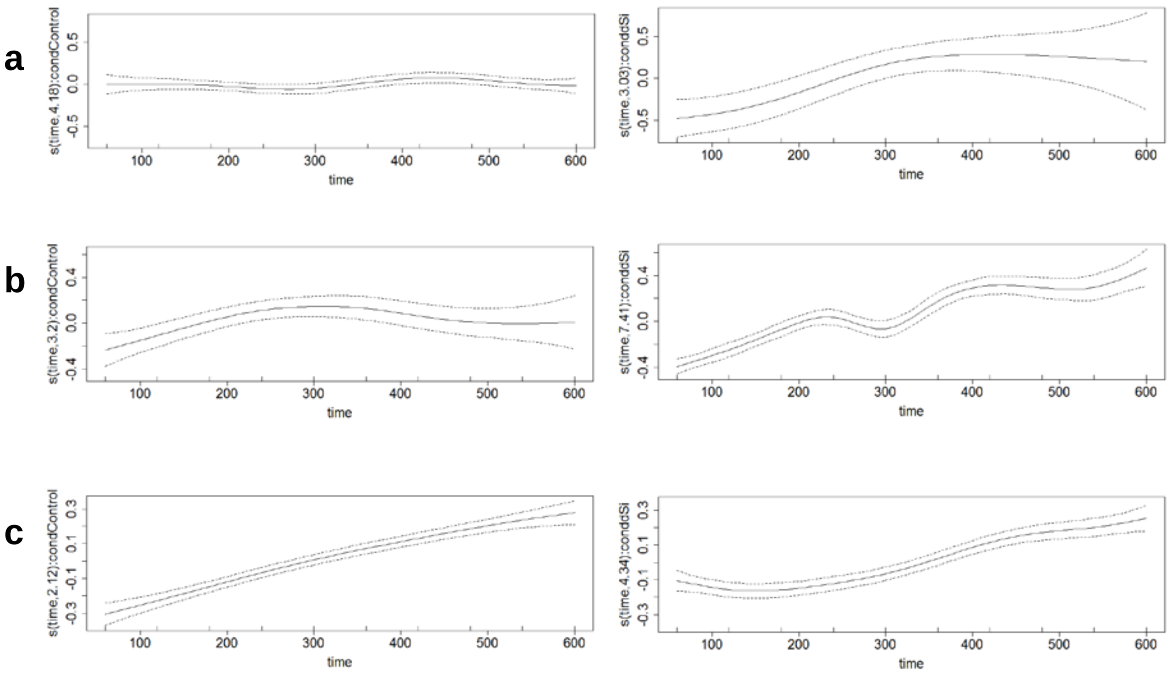

Supplementary Figure 3. Fitted cubic splines of cell speed for control (condCon) and dSi (condSi) treatment for bins A-C over T=600s. The data is fitted using smoothing splines which place a knot on each data point over time (s(time)). The optimal number of smoothing knots (i.e. the number of connections for each data point) of the fitted model is shown on the y-axis. GAMM models centers the mean of each treatment to 0. **a,** For Bin A, speed of cells exposed to dSi treatment were significantly increasing ($p=8.27^{-8}$) compared to control ($p = 0.176$). The smoothed plots showed that cell speed increased over time in the dSi treatment while a more random pattern mostly centered around 0 could be seen in the control treatment . **b,** For Bin B, both dSi and control gave statistically significant smooth terms with dSi giving a stronger effect ($p=<2^{-16}$) than control ($p=0.00259$). **c,** On the other hand, the trend on Bin C was the same for both control and dSi treatments.

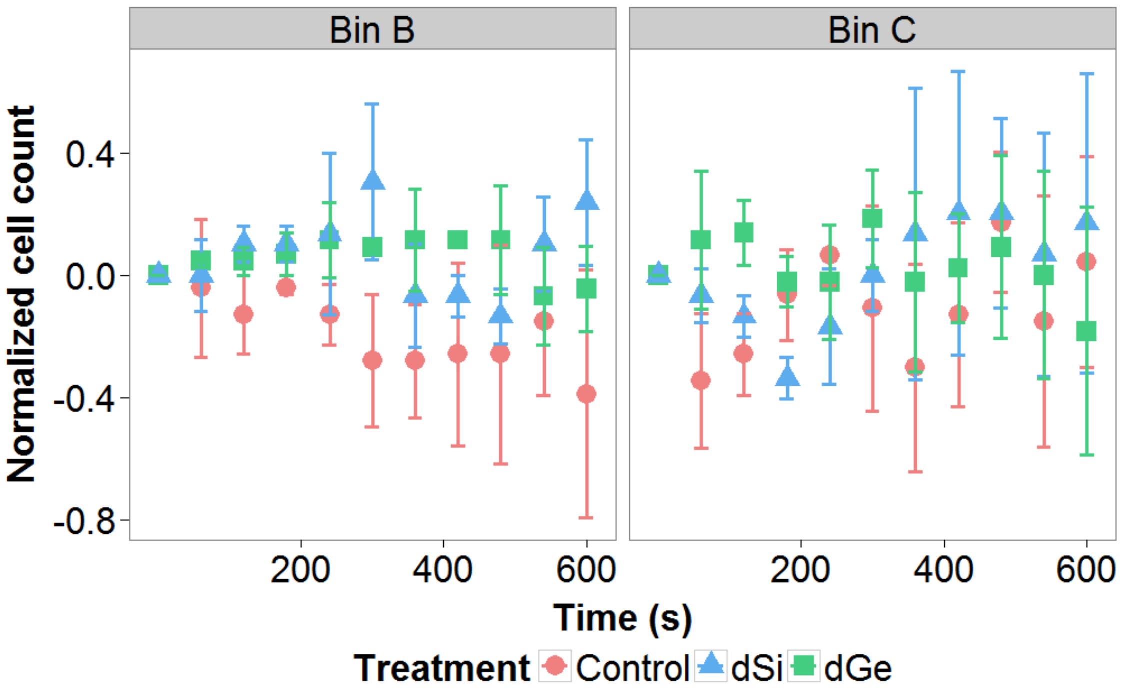

Supplementary Figure 4. Plotted normalized cell counts from every 60 s for Bins B and C of the substrate specificity testing. For normalization of the starting point to 0, we subtracted the standardized cell count on each time point to the value at T = 0 s. A value of 0 indicates that the cell density is equal to the mean. Positive values indicate a cell density higher than the mean. We observed no significant differences on both bins.

Supplementary Table 1: Cell speeds of starved and non-starved cells were compared before and after 1h addition of dSi (+dSi) or blank addition. Pairwise comparisons via Tukey's HSD were done after fitting the data with linear mixed effects model. Starved cells significantly move faster than non-starved cells. Addition of dSi to starved cells significantly increased the speed while addition of blank showed no effect. Moreover, when dSi is added to starved cells, the mean speed is almost the same when dSi is added to non-starved cells. Blank addition is significantly different from starved and non-starved cells with dSi addition.

Pair-wise comparisons	Estimate	Std. Error	z value	Pr(>|z|)
**Before dSi addition**				
**starved – non-starved**	0.4036	0.0250	16.165	<1e-04 ***
**After dSi addition**				
**starved+dSi – starved**	-0.5223	0.0277	-18.865	<1e-04 ***
**starved+blank – starved**	0.0452	0.0338	1.338	0.655
**starved+dSi - starved+blank**	-0.4772	0.0306	-15.60	<1e-04 ***
**starved+dSi - non-starved+dSi**	-0.0070	0.0201	-0.346	0.997
**starved+blank - non-starved+dSi**	0.4702	0.0280	16.802	<1e-04 ***
**non-starved – starved+blank**	0.3584	0.0281	12.753	<1e-04 ***

Signif. codes: 0 '***' 0.001 '**' 0.01 '*' 0.05 '.' 0.1 ' ' 1 (Adjusted p values reported -- single-step method)

Supplementary Table 2. Linear mixed effects model on count data (Control vs. dSi) for individual bins.

	numDF	denDF	F-value	p-value
**Bin A**				
**(Intercept)**	1	58	3.706	0.059
**treatment**	1	4	2.190	0.213
**Time**	1	58	2.356	0.130
**treatment:Time**	1	58	1.992	0.164
**Bin B**				
**(Intercept)**	1	58	0.0140	0.906
**treatment**	1	4	3.360	0.141
**Time**	1	58	0.042	0.838
**treatment:Time**	1	58	9.363	0.003
**Bin C**				
**(Intercept)**	1	58	0.507	0.479
**treatment**	1	4	5.372	0.081
**Time**	1	58	0.304	0.584
**treatment:Time**	1	58	7.571	0.008

Supplementary Table 3. Spline terms of general additive mixed effects model on cell speed (Control vs. dSi) for all bins.

	edf	Ref.df	F	p-value
**Bin A**				
**s(Time):condCon**	4.181	4.181	1.756	0.176
**s(Time):condSi**	3.035	3.035	12.075	8.27^{-8}
**Bin B**				
**s(Time):condCon**	3.200	3.200	4.479	0.003
**s(Time):condSi**	7.413	7.413	26.199	$<2^{-16}$
**Bin C**				
**s(Time):condCon**	2.117	2.117	73.50	$<2^{-16}$
**s(Time):condSi**	4.337	4.337	30.41	$<2^{-16}$

Supplementary Table 4. Linear mixed effects model on sine angle data (Control vs. dSi) for individual bins.

	numDF	denDF	F-value	p-value
**Bin A**				
**(Intercept)**	1	2004	0.192	0.662
**treatment**	1	17	0.140	0.713
**Time**	1	2004	9.754	0.002
**treatment:Time**	1	2004	0.702	0.402
**Bin B**				
**(Intercept)**	1	6190	0.309	0.578
**treatment**	1	35	0.268	0.608
**Time**	1	6190	4.043	0.044
**treatment:Time**	1	6190	0.261	0.609
**Bin C**				
**(Intercept)**	1	9792	0.168	0.682
**treatment**	1	48	2.887	0.096
**Time**	1	9792	22.62	<0.0001
**treatment:Time**	1	9792	0.666	0.414

Supplementary Table 5. Linear mixed model on sum distance (Control vs. dSi) for all bins.

	DF	Sum Sq	Mean Sq	F value	p value
**Bin A**					
**treatment**	1	189.98	189.98	17.42	<0.0001
**Time**	1	28.105	28.105	2.577	0.128
**treatment:Time**	1	94.442	94.442	8.658	0.010
**Bin B**					
**treatment**	1	13460	13460	105.04	1.94^{-8}
**Time**	1	5804.3	5804.3	45.295	4.88^{-6}
**treatment:Time**	1	2792.4	2792.4	21.791	<0.001
**Bin C**					
**treatment**	1	4691.6	4691.6	10.754	0.005
**Time**	1	2423.9	2423.9	5.5557	0.031
**treatment:Time**	1	1987.5	1987.5	4.5556	0.049

Supplementary Table 6. Linear mixed effects model on count data for substrate specificity (Control vs. dGe vs. dSi).

	numDF	denDF	F-value	p-value
**Bin A**				
**(Intercept)**	1	87	0.3857	0.5362
**treatment**	2	6	26.025	0.0011
**Time**	1	87	0.6343	0.4279
**treatment:Time**	2	87	11.745	<.0001
**Bin B**				
**(Intercept)**	1	87	0.0726	0.7882
**treatment**	2	6	1.3802	0.3213
**Time**	1	87	0.8504	0.3590
**treatment:Time**	2	87	1.382	0.2566
**Bin C**				
**(Intercept)**	1	87	0.004	0.9475
**treatment**	2	6	0.046	0.9551
**Time**	1	87	0.025	0.8745
**treatment:Time**	2	87	0.387	0.6806

Supplementary Table 7. Linear mixed effects model on count data (Control vs. different dSi concentrations).

	numDF	denDF	F-value	p-value
**(Intercept)**	1	116	9.896	0.0021
**treatment**	3	8	5.677	0.0221
**Time**	1	116	19.47	<.0001
**treatment:Time**	3	116	12.51	<.0001
